# Two-Stage Probability-Enhanced Regression on Property Matrices and LLM Embeddings Enables State-of-the-Art Prediction of Gene Knockdown by Modified siRNAs

**DOI:** 10.3390/ijms262411791

**Published:** 2025-12-05

**Authors:** Ivan Golovkin, Denis Shatkovskii, Nikita Serov

**Affiliations:** Center for Artificial Intelligence in Chemistry, ITMO University, 191002 Saint-Petersburg, Russia; golovkin2003@list.ru (I.G.); shatkovsky@scamt-itmo.ru (D.S.)

**Keywords:** machine learning, siRNA, gene knockdown

## Abstract

Six small interference RNAs (siRNAs) have been approved as therapeutics since 2018 making them promising nanosystems due to selective gene knockdown activity. siRNA design is complex due to various factors, where the chemical modifications are crucial to improve its half-life and stability. Machine learning (ML) enabled more efficient analysis of siRNA data, moreover predicting efficacy and off-target effects. This work proposes a novel pipeline for predicting gene knockdown activity of chemically modified siRNAs across the whole range of activities leveraging both descriptors of siRNA chemical composition-aware property matrices and large language model (LLM) embeddings for target gene encoding. Several general-purpose and domain-specific fine-tuned LLMs were benchmarked on the target task, where the Mistral 7B general-purpose model slightly outperformed even the models pre-trained on genomic data. Proposed two-stage probability-enhanced model successfully mitigates data imbalance towards moderate-to-high active constructs and achieves state-of-the-art (SOTA) quality with R^2^ = 0.84 and a RMSE = 12.27% on unseen data, where the probabilistic outputs of classifiers trained with F-scores up to 0.92 were used for regression model supervision. Moreover, leave-one-gene-out (LOGO) experiments show that the model is able to extrapolate on unseen genes, which further shows representativeness of siRNA features and gene embeddings. By filling the gap in the field of advanced chemical composition-aware siRNA design, our model aims to improve the efficacy of developed siRNA-based therapies.

## 1. Introduction

**Figure 1:**
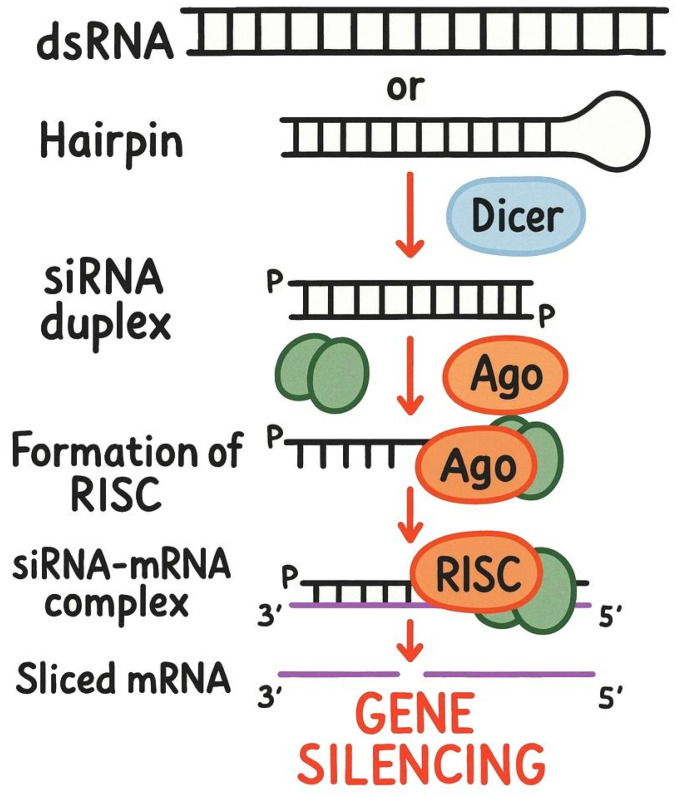
Schematic depiction of small interfering RNA (siRNA) mechanism of gene knockdown activity. Schematic created with DALL-E 3 and edited by the authors.

Gene knockdown is a modern technique in genetics and molecular biology used to investigate gene functions and their roles in disease pathogenesis. This method temporarily inhibits or reduces the expression of specific target genes, allowing cells or model organisms to recover and eventually return to previous expression levels. Unlike knockout techniques and mutation/knock-in approaches that create permanent changes to genomic DNA, gene knockdown primarily interferes with naturally occurring RNA molecules, including matrix RNA (mRNA) and non-coding RNA (ncRNA), without direct host DNA editing. siRNAs have received a lot of attention [1] because of their high specificity towards the target mRNA sequences, enabling effective gene silencing without significantly affecting other genes (Figure 1) [2,3]. Since 2018, six therapeutic siRNAs have been approved for the treatment of multiple human diseases; thus, siRNAs hold great promise as broad RNA-based therapeutics [4]. Moreover, the design of siRNAs is essential for understanding gene regulation in molecular biology. Current methodologies can be categorized into experimental and heuristic approaches. Experimental methods include synthetic biology for creating novel siRNAs targeting specific genes [5] and CRISPR/Cas9 technology for precise gene editing [6]. Cloning and expression systems help assess the functionality of designed siRNAs, while next-generation sequencing (NGS) provides insights into siRNA expression across different conditions and tissues. Heuristic approaches rely on intuitive practices, such as sequence analysis to identify conserved motifs among species and developing models to optimize siRNA-target interactions. Therefore, siRNAs have become a powerful tool in various research areas, including predicting efficacy and off-target effects [7,8], uncovering cellular processes and disease mechanisms, elucidating the role of siRNA in diseases [9], and advancing siRNA delivery and drug discovery [10].

siRNA basic design and synthesis are straightforward unless modifications are used to improve performance. This provides significant improvements, but makes the manufacturing process more complex. Unlike DNAzymes, which may require complex optimization, siRNA provides a more user-friendly approach for transient gene suppression, making them ideal for functional studies. Additionally, while shRNA can lead to toxicity due to persistent expression, siRNA allows for controlled and temporary knockdown of gene expression [11]. Compared to CRISPR, siRNA serves as a less invasive method for studying gene function while avoiding unintended genetic modifications [12]. In addition, siRNA directly affects innate immunity and cytokine production [13]. The physical structure of siRNA is crucial for target inhibition. The typical length of siRNA ranges from 20 to 25 nucleotides. Longer siRNAs can exhibit increased stability but may have reduced specificity and binding efficiency to their target mRNAs. Mismatches between siRNA and target mRNA can significantly affect siRNA function. They can decrease binding affinity, especially when located at critical positions, leading to reduced repression of target genes and unintended protein expression [14]. In addition to the utility of siRNAs composed of naturally occurring nucleotides, some chemical modifications were shown to further enhance siRNA stability against nucleases and improve target specificity [15,16,17,18]. Modifications can increase resistance to degradation and alter base pairing properties, efficiently suppress immunostimulatory siRNA-driven innate immune activation, enhance activity and specificity, and reduce off-target-induced toxicity [19].

Since theoretical modeling of these systems remains computationally complex, current computational methods often leverage bioinformatics and ML to design efficient siRNAs. For instance, RNAfold [20] and mFold [21] are used to predict the secondary structures of RNA sequences, including siRNAs, a step vital for understanding their stability and interactions. ML approaches have been extensively used to predict siRNA efficacy, allowing researchers to prioritize candidate siRNAs with higher likelihood of success, while identifying potential off-target effects helps ensure the safety of therapeutic applications. Early deep learning work already showed that combining local sequence context with thermodynamic descriptors (e.g., via convolutional networks) can improve knockdown prediction compared with hand-crafted rules [22], motivating today’s broader move toward representation learning. More recently, DeepSipred combined sequence-context features with biophysical/thermodynamic properties in a deep-learning pipeline for siRNA inhibition prediction, illustrating the continued value of hybrid feature sets [23]. ML was also used to unveil the intricate cellular processes involving siRNAs, including their uptake mechanisms and interactions with other cellular components, allowing for targeted delivery strategies and improved understanding of siRNA-mediated gene silencing [24]. Moreover, ML models are increasingly employed to elucidate the role of siRNAs in specific diseases, providing insights into potential therapeutic interventions.

Despite all these advancements, quantitative modeling of modified siRNA efficacy with high precision has remained a challenge. In this work, we fill this gap by introducing a two-stage probability-enhanced regression model with state-of-the-art (SOTA) performance operating on siRNA and gene sequence context as well as on the chemical composition of individual nucleotides. The model was trained on >3400 experimental data points with 2797 unique siRNA designs, 37 unique chemical modification types targeted at 47 genes. The model achieved an R^2^ of 0.84 and RMSE of 12.27% on unseen test samples, outperforming all the current models including deep learning (DL) models. These findings allow researchers to precisely model siRNA activity as well as modification effects crucial in practical application of these chemical systems.

## 2. Results

### 2.1. Data Collection and Engineering

#### 2.1.1. Data Extraction and Curation

The dataset was obtained from the siRNAmod database [25], encompassing information on chemically modified siRNAs, including their sense and antisense sequences, chemical modifications, their positions as well as knockdown efficacy. For a full description of each experiment, additional parameters such as the method of siRNA activity assessment, siRNA melting point, the target gene, the cell type or organism used for transfection, the transfection method, post-transfection duration, and other experimental parameters were collected (see Section 3.1). The original database contained approximately 5000 entities and information about two nucleotide sequences, the types of modifications, and their positions, as well as gene knockdown efficacy. The initial dataset has gone through rigorous cleaning and standardization procedures to ensure data quality and consistency (for details see Section 3.1). In addition to standard data cleaning, chemical modifications were manually processed to produce modified nucleotide SMILES, where a total of 37 unique modifications were extracted (Table S12). Certain modifications exhibited ambiguity or incorrect chemical structures, making their explicit assignment to specific nucleotide positions challenging. These modifications were consequently excluded from the final dataset. Samples with missing values were deleted, resulting in a final dataset with a total of 3435 processed entities.

#### 2.1.2. Feature Engineering

RDKit [26], PyBioMed [27], and CDK [28] chemical descriptors were used to describe both sense and antisense siRNA sequences. RDKit emphasizes a comprehensive range of descriptors, including physicochemical properties, e.g., partition coefficients (LogP), topological polar surface area (TPSA), molecular weight, total number of aliphatic rings, valence atoms, among others. At the same time, PyBioMed includes specialized descriptors for bioactivity, such as pharmacophore features and protein-sequence-based descriptors, which are less prevalent in RDKit. In contrast, CDK includes more detailed and explicit 3D descriptors related to molecular shape and geometry; therefore, these descriptors capture additional groups of molecular properties. To characterize target gene diversity, the corresponding nucleotide sequences were extracted from the NCBI database and later processed by LLMs to generate embeddings, which yielded a total of 2642 processed entities.

### 2.2. Data Analysis

#### 2.2.1. Exploratory Data Analysis

**Figure 2:**
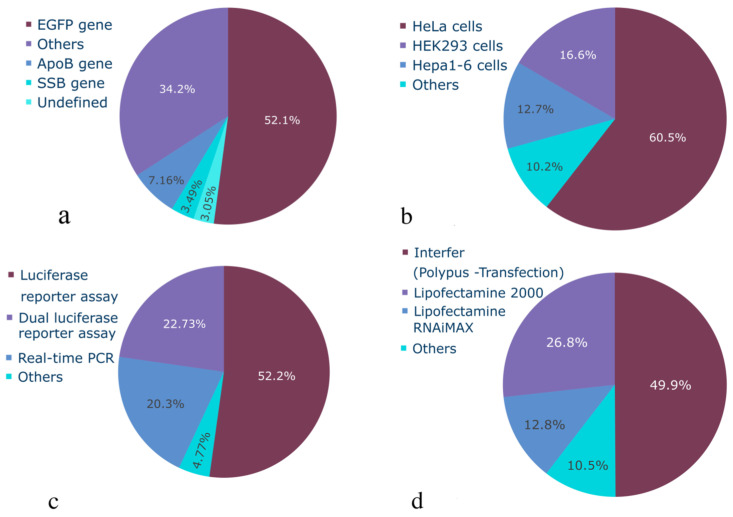
Statistical characterization of categorical variables of siRNA dataset: (**a**) target genes used, (**b**) cell or organism used, (**c**) activity measurement method, (**d**) transfection method.

**Figure 3:**
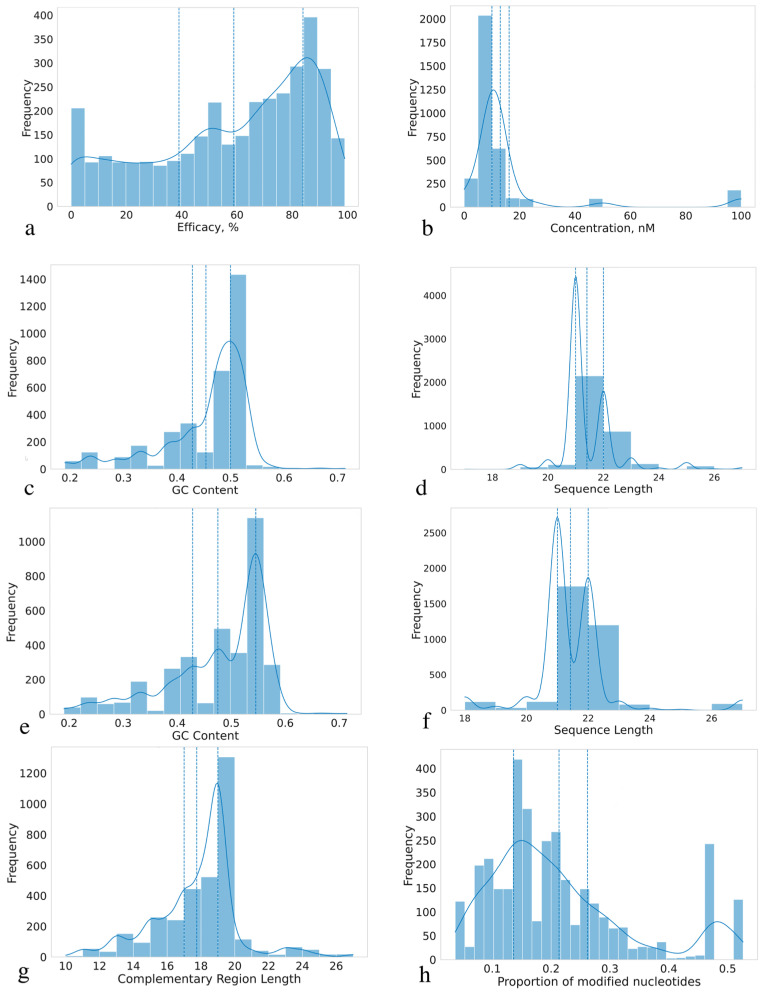
Statistical characterization of siRNA dataset: (**a**) efficacy, %, (**b**) siRNA concentration, nM, (**c**) GC content of sense sequences, (**d**) sense sequences length, (**e**) GC content of antisense sequences, (**f**) antisense sequences length, (**g**) complementary region length, (**h**) proportion of modified nucleotides per siRNA. Doted curves stand for probability density. Vertical doted lines stand for 25th and 75th percentiles.

This section describes statistical properties of siRNAs, examining their length distributions, GC content, modifications, and complementary regions, as well as investigating categorical parameters. By analyzing these characteristics, we aim to assess whether the collected dataset is sufficiently representative for ML model development and determine what parameters influence siRNA efficacy on gene knockdown. All distributions of the variables are presented in Figure 2 and Figure 3 (boxplots for continuous features are presented on Figure S2). The distribution of siRNA concentrations used in the experiments reveals that most concentrations are clustered around 0–20 nM, with a particularly high frequency observed at approximately 10 nM (Figure 3b). Based on the available experimental data, 20 nM and lower concentrations can be considered the optimal concentration range due to notable off-target effects above this threshold [29]. The effective siRNA concentration in cells after transfection starts at 0.1 nM and reaches a conditional plateau around 10 nM; therefore, values from 0.1 to 20 nM can be considered sufficient and optimal for observing knockdown efficacy [30], although for transfection of certain cell types, the optimal concentration can be up to 30 nM [31]. Therefore, the dataset can be considered representative, containing siRNAs in a wide range of minimal effective concentrations (MEC), which is crucial to avoid bias in future algorithms.

The distribution of gene knockdown efficacy percentage is shown in Figure 3a. The dataset exhibits a slight imbalance towards highly effective siRNAs, with a median efficacy higher than the mean, indicating a right-skewed distribution peaking around 50%, which is likely due to the frequent reporting of IC50 values. However, the inclusion of a number of low-efficacy siRNAs (<50%) mitigates this imbalance and should allow for the development of unbiased predictive models. Therefore, the collected dataset encompasses a wide range of samples with varying efficacy. This breadth ensures the dataset’s completeness and enables future ML models to effectively handle siRNAs with varying knockdown efficacy potential. The distribution of categorical variables is presented in Figure 2. The dataset predominantly comprises model experiments on the GFP gene knockdown in HeLa cells. While this dataset provides valuable insights into siRNA functioning and is suitable for model development, the accumulation of unbiased experimental data across a broader range of genes and cell lines remains a significant challenge.

#### 2.2.2. Correlation Analysis

To assess the inter-feature relations as well as the dependence of the target value on the numerical parameters of the experiment, Pearson’s correlation analysis was performed (Tables S1 and S2, respectively). Neither siRNA concentration nor time after transfection was highly correlated with knockdown efficacy indicating an expected absence of linear dependencies. The most correlated descriptor features were also identified, as discussed below. The proportion of sp^3^-hybridized carbon atoms indicates a molecule’s saturation and non-planarity, crucial for flexibility and binding ability. The indices, based on valence connectivity, reflect molecular branching and complexity. The number of aliphatic rings counts the number of non-aromatic rings, and the number of saturated rings specifically identifies the count of rings with only single bonds. Together, these descriptors offer insights into a molecule’s topology, shape, and chemical characteristics, demonstrating low correlations with the target value in the range of 0.22–0.27. Spearman’s rank correlation test was used to calculate the correlation with categorical variables (Table S2), also showing little correlation with the knockdown efficacy. Individually, no single attribute correlates significantly with the target value, which shows the need to build non-linear models that estimate the efficacy on a set of parameters. This verification indicates only the absence of linear or non-linear monotonic dependencies, although more complex relationships cannot be excluded based on these tests.

#### 2.2.3. Sequence Analysis

The majority of sequences have a conservative length of 21–22 nucleotides, with this length accounting for more than 93% of the sequences (Figure 3d,f). siRNAs are sensitive to degradation by nucleases, and the typical length of 21–22 nucleotides provide an optimal balance between binding affinity, stability, and susceptibility to degradation. siRNAs that are too short are more prone to degradation, and siRNAs that are too long may have slower and less efficient transport into the cell. The length of the sequences also affects the stability of the complex with mRNA and the interaction with RISC. We observed a number of sequences in hybrids with lengths below 18 nucleotides (1 hybrid) and above 25 nucleotides (24 hybrids). However, these outliers are relatively few in number and do not significantly impact the overall distribution. Long hybrids were predominantly highly efficient, but in some cases the efficacy was close to zero, which indicates that such systems are understudied despite their controversial and non-trivial activity profiles.

Distributions of GC content in both sense and antisense sequences are shown in Figure 3c,e. The GC content analysis revealed a noticeable peak around 50% (corresponding to duplex melting temperature around 70–73 °C) in both sense and antisense sequences, suggesting a prevalence of sequences with approximately equal proportions of guanine-cytosine (G-C) and adenine-thymine/uracil (A-T/U) base pairs. GC content directly affects the melting point and degradation by nucleases, where higher values provide better stability and resistance to nucleases. The most common content is 0.4–0.5, which is also observed in our dataset, where the melting point for RNA-RNA hybrids is predominantly in the range of 67–73 °C. Based on Figure 3g, which depicts the distribution of complementary regions, most of them are centered on 21 base pairs. Minor peaks were observed at 18–20 and 22–27 base pairs, but their frequencies were considerably lower compared to the dominant peak. Notably, the sequence hybrids have melting points in the range of 67 to 73 °C (see Section 3.1). This range depends on GC content, the number of mismatches in the hybrid, and the length of the chains. Melting point analysis is important for understanding the possibility of forming a stable complex with RISC, but not so strong as to interfere with chain unraveling. Temperatures significantly above 70 °C are considered undesirable: off-target effects increase due to non-specific binding [32]. The boxplots shown in Figure 3h illustrate the distribution of the proportion of modified nucleotides per sequence pair. Selecting the optimal number of modifications is also an important task for increasing the efficacy of gene knockdown. The dataset has a fairly wide range of siRNAs with different percentages of modified nucleotides, which supports improved predictive performance and model generalizability.

#### 2.2.4. Mismatch Effect on Gene Knockdown

To assess the complementarity of the sequences, we analyzed them for the presence of mismatches. Knockdown efficiencies for the longest matching region are presented in Figure S1. The dataset contains 547 entities with mismatches (16% of the entire dataset). The efficacy here was normalized by the siRNA concentration C_siRNA_ and the incubation time t_incubation_ (Equation (1)) for comparison purposes, where larger values indicate greater efficacy. To enable the direct comparison of siRNA efficacy across experiments performed at different concentrations and incubation times, a linear normalization was applied using Equation (1). The efficacy normalization was performed solely for visualization and distribution analysis purposes and was not used as the target variable in the further work due to the sigmoidal dose–response and time-kill kinetics observed in biological systems.

(1) $${Efficacy}_{normalized}=lg(1+\frac{Efficacy}{C_{siRNA}*t_{incubation}})$$

Based on Figure S1, we can say that the length of the complementary region is a weak predictor of normalized knockdown efficacy. The observed U-shaped relationship indicates a complex dependence between complementary region length and its effect on normalized efficacy. Generally, higher normalized efficacy is associated with shorter (short matches) and longer complementary regions (long matches), while the medium complementary length tends to exhibit lower efficacy, which is a non-trivial observation. Interestingly, having two mismatches in hybrids resulted in a greater efficacy than having only one mismatch, whereas the presence of mismatches in the siRNAs often resulted in lower efficacy, except when pairs of mismatches were found close to the edges of the chains. In general, mismatches lead to greater variation; however, the opposite effect may also occur, as mismatches can increase thermodynamic stability [33]. If there is a mismatch between the siRNA and the target mRNA, the duplex becomes less stable. This can lead to an increased likelihood of knockdown of a non-target gene. Our results strengthen the theory of Ahmed et al. [14] that 1 or 2 mutations in complementary regions can be used to reverse the efficacy of an siRNA from ineffective to effective and vice versa.

#### 2.2.5. Modifications Influence on siRNA Efficacy

The most common modifications occur in the sugar component, including methylation of 2′ oxygen (2-O-methyl or 2-methoxy), locked nucleic acids (LNA), and 2′-fluoribose, with the only base modification found in the dataset being deoxythymidine. Additionally, there is one modification for the entire nucleotide, known as inverted abasic. Modifications of the sugar bases, such as 2′-O-methylation and LNA, primarily aim to enhance stability, improve resistance to enzymatic degradation, increase binding affinity and specificity towards target RNA [34]. Substitution of U with deoxythymidine also affects the affinity of the modified siRNA to the target RNA. This modification often results in increased resistance to nucleases, leading to a longer half-life and enhanced efficacy of gene knockdown. The inverted abasic modification removes the purine or pyrimidine base from the nucleotide, leaving only the sugar-phosphate backbone, and it also affects the mechanism by which the sugar is attached. This alteration can significantly diminish the affinity of siRNA for its target RNA since base-pairing cannot occur at this site. While this modification is rare and typically located at the ends of sequences, its presence in the central part of the sequence can drastically reduce knockdown efficacy. Despite this drawback, inverted abasic modifications are employed to enhance overall stability, which remains a primary reason for their inclusion. The impact on knockdown efficacy varies: while some modifications can enhance gene silencing, others might reduce it depending on the specific target and the position of the modification within the sequence [35].

**Figure 4:**
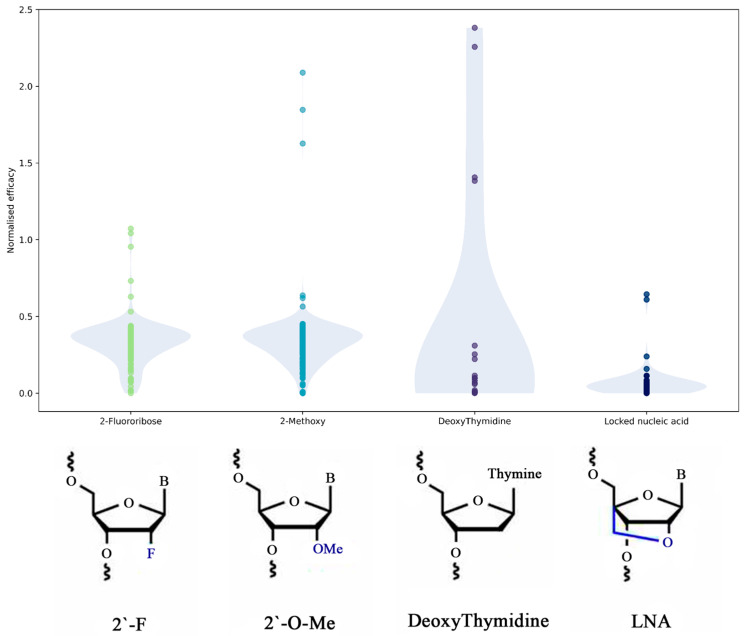
Distribution of normalized efficacy of sequences depending on the modification type (probability density plotted in opaque colors).

The effect of the most common modifications on the efficacy of siRNA designs was further studied. Figure 4 shows the normalized efficacy for sequences that contain only one type of modification. These distributions indicate that, to achieve the most efficient gene knockdown, several types of modifications should be used in RNA hybrids. The 2′-fluoribose and 2′-O-methylmodifications perform best alone. The methylation of 2′ oxygen was found to improve serum stability, binding affinity, as well as reduce the innate immune response, whereas 2-fluoribose has been reported to have limited nuclease resistance as compared to other 2′-ribose modifications. To increase chain stability and efficacy, methylation of 2′ oxygen is a more favorable choice than LNA, although in some cases and combinations with other modifications, LNA may be a preferable option. LNA aims to improve endonuclease stability and reduce innate immune responses; therefore, it may be used with 2′-fluoribose to achieve an effect similar to 2′-O-methylation. However, adding LNA also massively increases duplex stabilization and leads to backbone changes [36]. Deoxythymidine should only be used in combination with other modifications, as individual effects are the least obvious based on our dataset.

Our understanding of the target value, the types of modifications, and the correlations between the initial parameters of our experiments and the target value has enabled us to identify specific descriptors for future analysis and model development. Additionally, a wide range of target genes and significant variability in siRNA sequences are observed for the dataset. This comprehensive knowledge allows us to perform feature selection effectively and prepare the necessary pipeline for the development and use of our predictive model.

### 2.3. Predictive ML Models

To go beyond categorical description of genes and provide further model flexibility towards unseen genes, nucleotide sequences for the target gene were extracted using the NCBI database, with embeddings obtained for the sequences using general-purpose (Mistral) and domain-specific (MistralDNA, HyenaDNA) pre-trained LLMs. All final predictive models run on RDKit descriptors because of the best combination of metrics on the test and training datasets, as well as the lowest dimensionality (Table 1, and for details see Section 2.3.2).

#### 2.3.1. siRNA Classifiers

##### Binary Classifier

**Figure 5:**
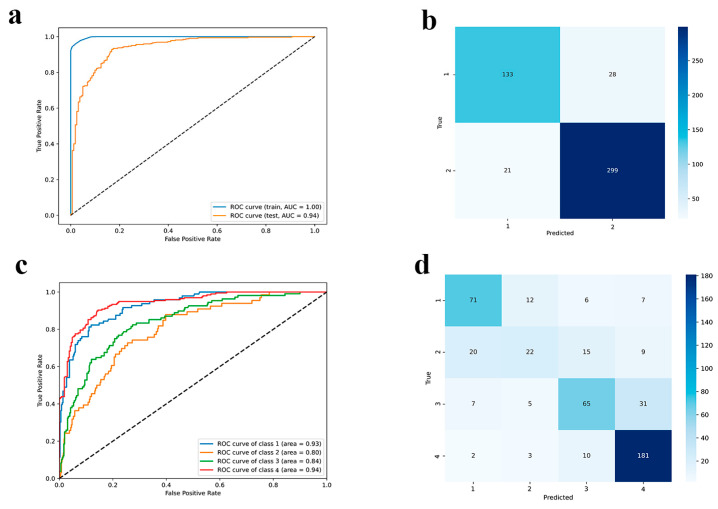
Performance represented with (**a**,**c**) ROC-AUC curves and (**b**,**d**) confusion matrix on the test set for binary and multiclass classifiers, respectively.

Given the complexity of accurately predicting siRNA activity in a quantitative manner, we initially adopted a two-stage approach. A classification model was first employed to efficiently identify potentially active siRNAs, addressing the dataset’s imbalance towards highly effective candidates which could bias a regression model. This initial filtering step allows us to focus a more accurate regression model on a refined subset of promising candidates, leading to more reliable performance estimates for truly active siRNAs. The results of the binary classification model are summarized in Figure 5a,b and Table S3.

Here, the data was divided into two ranges: the first contains samples with efficacy values between 0% and 45%, while the second contains values between 55% and 100%. Since the majority of misclassified samples occur at boundary regions, some samples bearing intermediate efficacy from 45 to 55% were removed for better class separation. For binary classification, we used 75 features from descriptors and embeddings and 5 variables from the original dataset (see Section 3.3). Precision, recall, and F1-score values for both class 1 and class 2 are high, signifying excellent performance in correctly identifying instances belonging to each category (Table S3). The model maintains a high accuracy of 0.90 on the test set, suggesting a good ability to generalize to unseen data. While the performance slightly drops compared to the training set, the precision, recall, and F1-score values for both classes remain above 0.83, demonstrating strong predictive power. Receiver Operating Characteristics (ROC) curve and Area Under the Curve (AUC) equal to 0.94 show strong predictive performance and high area under the curve (Figure 5a), outperforming the recent classification models [37,38]. The confusion matrix reinforces the belief that low-performing siRNAs are less represented in the dataset, so this affects the slightly lower predictive accuracy; high-performing samples are predicted better (Figure 5b). In general, classification models perform strongly on new, previously unseen data. Our model demonstrated excellent performance on a simple binary classification task. Therefore, we extended our analysis to a more complex scenario characterized by the presence of 4 imbalanced classes for deeper categorization.

##### Multiclass Classifier

The four data classes cover the following ranges: 0–23% (first class, including extreme values), 27–48% (second), 52–73% (third), and 77–100% (fourth). For the last three classes, the upper threshold has been included in each class. For multiclass classification we used 66 features from descriptors and embeddings and 5 variables from the original dataset (see Section 3.3). Results of multiclass classifier performance are shown in Figure 5c,d and Table S4.

The performance drops on the test set, with an accuracy of 0.73. The task became more complex and the metrics expectedly dropped; however, the quality is still fairly good on three out of four classes, where the best performance is on classes 1 and 4, i.e., for siRNAs with high-efficacy, which are most represented in the dataset. The precision, recall, and F1-score values are also lower for all classes, indicating a decline in the model’s ability to generalize to unseen data. Being relatively abundant, classes 1 and 4 were predicted well by the model (Table S4). Problems with the assignment of the second and third classes are related to the large number of values with an efficacy of 50%, which do not allow the model to accurately assign them to a class. The results for binary classification are more homogeneous when compared to multiclass classification because of the less pronounced separation in the data and because of the elimination of boundary values between classes for clearer model performance.

#### 2.3.2. Knockdown Efficacy Quantitative Prediction

##### Descriptors and Models Comparison

To investigate the feasibility of quantitatively predicting siRNA efficacy, we moved beyond the initial classification approach. Several descriptor types were numerically compared to identify the optimal features for a regression model (Figure 4) aimed at predicting the efficacy of siRNA-based therapies (Table 1).

**Figure 6:**
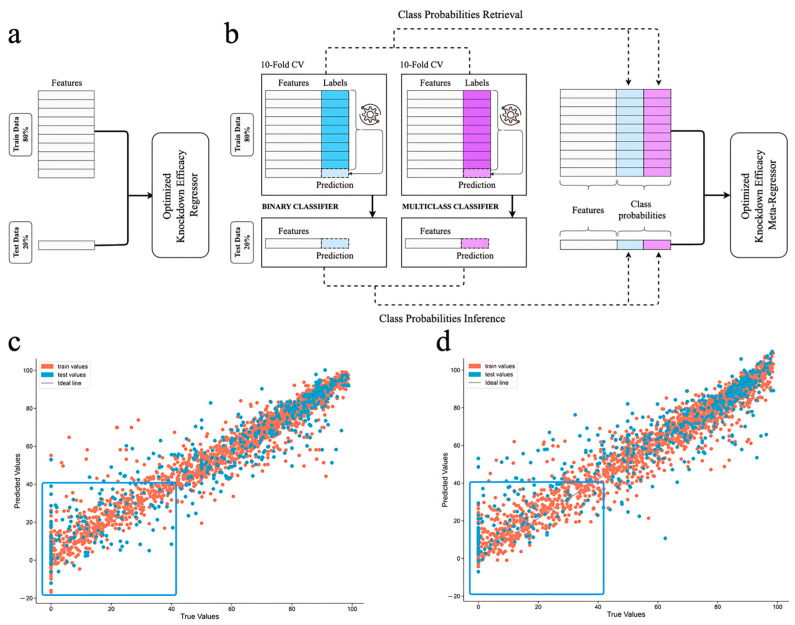
Quality of (**a**,**c**) vanilla regression and (**b**,**d**) two-stage probability-enhanced regression model performance in quantitative gene knockdown efficacy prediction on training and test data. Probabilities of all categories (blue and violet columns) were obtained from both classifiers. The gear symbol indicates the algorithms were optimized via feature selection and hyperparameter tuning at each stage of 10-fold CV. Red dots stand for the train samples, blue—for test samples. Blue box highlights the differences in models’ performance on low-activity samples.

RDKit, PyBioMed, and CDK are powerful libraries for generating descriptors of siRNAs. Specialized nucleotide descriptors such as PseKNC or NUPACK-derived features were excluded from the comparison as they lack the capacity to explicitly account for chemical modifications in the nucleotide structure, which represents a significant limitation, resulting in the loss of important chemical information. RDKit is a chemical modelling library that excels at analyzing and manipulating molecular structures. Descriptors focused on physicochemical and topological properties were chosen for use in our model. They showed similar performance to PyBioMed, thus, the descriptors for the final model were chosen according to other parameters. RDKit descriptors had lower dimensionality, were computed faster, and contained information about the chemical structure of the sequences, thus providing greater interpretability. We obtained a consistent number of descriptors, totaling 2322 separate parameters. RDKit was chosen for the final model as the one with lower dimensionality. The model was built using the LGBM model as the least overfitted (Table 1), in which the feature selection process resulted in 96 features used for training. In this way, we addressed collinearity problems, improved model performance and ease of interpretation, as well as reduced the chance of overfitting. Hyperparameter tuning was performed using Optuna, aiming to maximize the R^2^ score on the test set (for details see Section 3.3). The model achieved a notably high R^2^ score of 0.78 on the test set. The best parameters were achieved without using normalization at all stages of feature selection. To evaluate the performance of our model, we relied on test and training metrics RMSE, R^2^ (Figure 6c; performance over training iterations is shown in Table S5). Adjusted R^2^ for the test dataset was 0.74 (parameters for the model are reported in Section 3).

##### Probability-Enhanced Approach

To improve model predictive power, target gene LLM-derived embeddings from Mistral 7B were used instead of categorical labeling. Surprisingly, general-purpose Mistral 7B demonstrated superior performance compared to domain-specific pre-trained models MistralDNA and HyenaDNA when used to generate embeddings of target genes for a regression task and showed comparable results in both classification tasks (Tables S6–S8). The generalized Mistral slightly outperformed specialized HyenaDNA, which can be explained by higher dimensionality of Mistral embeddings. The next step was to repeat the pipeline from our previous model (Figure 6a) and evaluate it against a stacked generalization framework incorporating classification probabilities (Figure 6b).

For the final model, we adopted a probability-enhanced approach. This involves utilizing two of our classifiers along with a regression model that incorporates the labels generated during the classification stage based on 10-fold CV (Figure 6b). This approach (Figure 6d) outperformed the regular regression model (R^2^ increase from 0.81 to 0.84; see Figure 6c), showing state-of-the-art (SOTA) performance (Table 2). It should be noted that our comparison was limited to documented results from other models, as retraining on our own dataset was not feasible due to proprietary architectures, therefore hindering re-training on the same data splits. Consequently, the comparison was performed exclusively against the metrics reported by the original authors. Our R^2^ on the unseen data was 0.84, which proves strong predictive ability, and the model outperforms all existing shallow and DL regression models [38,39,40,41,42]. This model is more precise than the one based on gene label encodings and does not suffer from one important drawback, namely, it is able to predict efficacy for genes that were not used during the training process, through the use of nucleotide sequence embeddings. Equally important, the probability-enhanced approach allowed us to make better estimations for underrepresented low-active siRNAs (box in Figure 6d), and therefore shows better generalization capabilities. The SOTA performance of this model is achieved with early identification of inefficient siRNA designs using the classifiers described above. The possibility of data leakage was excluded by the application of 10-fold CV on the training set for the retrieval of probability labels needed for training the regressor, and the test sets were the same for both classifiers and the regressor. Therefore, a highly effective pipeline for quantitative prediction of gene knockdown efficacy across the whole range of activities was developed.

##### LOGO Experiment

Although the conventional model yields impressive performance, to further verify the robustness of its predictive ability, a stress test was performed. Specifically, an experiment designed to evaluate the impact of gene-specific information on the vanilla regression model revealed that removing the target gene feature from the training data of a baseline regression model did not significantly alter its predictive performance, which can be explained by the fact that both antisense and sense siRNA strands already contain information related to hybridization site. The Leave-One-Gene-Out (LOGO) experiment on the regression model demonstrated reduced predictive ability when evaluating genes not present in the training dataset (Table S11 with SSB, SOD2, and STAT1 genes selected as the most representative as both under- and overrepresented genes), although it should be noted that this result contradicts with the hypothesis that the whole gene context does not impact the model performance. These findings with the influence of stratification by gene suggest that information regarding siRNA-mRNA hybridization site already contained in siRNA descriptors is indeed an important component for the training set and for the model’s generalization capability on test data. Consequently, a significant portion of the genetic information is encoded within the siRNA features, which arises from their complementarity to specific target gene sequences.

## 3. Materials and Methods

### 3.1. Data Processing

The initial dataset was obtained from an online siRNAmod database [25]. Experiment metadata was collected using Requests 2.31.0 and bs4 Python 3.12 libraries. Dataset processing focused on removing outliers, anomalies and uninterpretable records, as well as on data standardization. All data preprocessing was performed with Python 3, mainly using the Pandas 2.1.2 and NumPy 1.26.1 libraries, as well as regular expressions via the re library. Sequences were checked to ensure proper nucleotide notation (four allowed nucleotide letters). Biological activity data was standardized to reflect the percentage of inhibition of the target gene. The concentration values were converted to nM where possible (excluding cases such as mg/kg/day). Values less than 0.01 and greater than 100 nM were also cleaned from the dataset. All samples containing EC50 or IC50 measurements were treated as indicating 50% efficacy at the reported concentration. Log-transformed titer values (in pfu/mL) were removed. Duration after transfection was parsed with regular expressions and converted to integer hours. To handle the nucleotide modifications, each nucleotide of the sequence was enumerated from left to right. Then, if the nucleotide did not contain any modifications, the SMILES representation was used in its unmodified form. Otherwise, the SMILES string of the corresponding nucleotide was manually constructed, taking into account its chemical modifications. For each SMILES string, descriptors were then calculated. All synonymous names of modifications were unified (Table S12). Mismatches were identified and quantified using the BioPython 1.80 library [43]. Alignment was performed using the pairwise2.align.localms function. Prior to alignment, reverse_complement() or reverse_complement_rna() was applied depending on the presence of T in the sequence. Mismatches for RNA hybrids were calculated according to the alignment. The formula was used

$$T_{melt}=81.5+0.41\times {guanine-cytosine(GC)}_{content,\;\%}-\frac{675}{{Length}_{hybridization}}-{Pair}_{mismatched,\;\%}$$

to calculate melting point, where gc_content_percentage was obtained using BioPython and mismatched_pair_percentage was defined as the ratio of mismatches to the hybrid region length.

### 3.2. Feature Engineering

RDKit 2023.9.1, PyBioMed 1.0, and CDK v2.0 toolkits were used to generate molecular descriptors from the SMILES representations of both sense and antisense sequences. RDKit provided 43 descriptors per molecule, PyBioMed generated 44, and CDK provided a larger set of 223 descriptors due to its broader coverage of molecular features, including geometry and bond connectivity. Descriptors were computed for each nucleotide in the sequence. To standardize the dimensionality across sequences of varying lengths, the longest sequence (27 nucleotides) was used as a reference, with shorter sequences padded with zeros. This resulted in 2322, 2376, and 12,042 descriptors per sense-antisense sequence pair for RDKit, PyBioMed, and CDK, respectively. The corresponding Python libraries were used to compute all descriptors. For the target gene variable, coding DNA sequences (CDSs) were retrieved from the NCBI database. Embeddings for these sequences were obtained using the Mistral 7B, MistralDNA, and HyenaDNA models. Vector representations were obtained by inputting the complete sequence into the model, where the last hidden state of the last token was used.

### 3.3. ML Models Development

The data were split at a ratio of 80/20 for training and evaluation, respectively, with random state set to 42 using sklearn. Metrics were also calculated using scikit-learn. The experimental variables—activity measurement type, cell or organism, transfection method, and target gene (when embeddings were not used)—were label-encoded. The feature selection protocol involved two stages: first, the top 100 features were ranked according to their importance derived from the training data only. Second, correlation analysis was conducted on the complete dataset; however, this analysis was restricted to inter-feature relationships, deliberately excluding correlations between features and the efficacy measure. This methodology ensures that no data leakage occurs during feature selection, where only features with a correlation greater than 0.95 with any other feature were removed. Therefore, this selection process was not stringent, resulting in a large number of parameters being retained. This step was used to prevent redundant features from remaining in the model. LGBM models and RDKit descriptors were used for both classification and final regression tasks. Each classification and regression model has a different final number of features. Data normalization was not used in classification and final regression models. RobustScaler was used for all parameters when comparing descriptor sets and regression models.

### 3.4. Classification Models

Two classifiers were built: (1) a binary classifier that distinguishes between two activity ranges: 0–45% and >55% and (2) a multiclass classifier that divides the data into four categories (0–23%, 27–48%, 52–73%, and 77–100%) with extreme values included. Parameter optimization was conducted using the Optuna library, involving 100 trials with a random state of 48. The goal of this optimization was to minimize (1 − accuracy). For binary classification, 80 features were obtained, together with 5 variables from the original dataset: siRNA concentration, transfection method type, cell or organism type, incubation time after transfection, activity measurement experiment type. For multiclass classification, 66 features were obtained, along with the same 5 experimental variables. For evaluating classification performance, we relied on metrics provided in the classification report: recall, precision, F1-score, accuracy, and the Matthews correlation coefficient (MCC). The hyperparameters of the multiclass and binary classification models are presented in Table S9.

### 3.5. Regression Models

To determine the best regression model and descriptor type, RDKit, PyBioMed and CDK descriptors were compared using 3 models: LGBM, Extreme Gradient Boosting (XGB), Random Forest (RF). Train and test RMSE, R^2^, 5-fold cross-validation R^2^ (CV R^2^) using KFold to evaluate models. Metrics were also calculated using sklearn. Comparisons were made using the label-encoded target gene. For the optimized non-embedded model, 96 columns were retained for the final training out of 2327 (Table S4). This included siRNA concentration, transfection method type, cell or organism type, incubation time after transfection, the type of activity measurement experiment, and the target gene name. Next, hyperparameter selection was performed. The hyperparameters tuning was performed using Optuna [44] with a total of 100 trials. The optimization was aimed at maximizing the test R^2^. The following parameters were optimized during this process (with a random seed set to 48): reg_alpha, reg_lambda, colsample_bytree, subsample, learning_rate, max_depth, num_leaves, min_child_samples, cat_smooth, max_bin, min_child_weight, boosting_type, scale_pos_weight. Regression model hyperparameters are presented in Table S10. For comparison with the probability-enhanced approach, the same pipeline was implemented using a predefined saved split (described below in Section 3.6). Additionally, an extra split was performed to create a validation dataset, using an 80/20 ratio of the training dataset. In this pipeline, 71 features were retained. Model hyperparameters are presented in Table S10.

### 3.6. Probability-Enhanced Approach

The dataset with embeddings, treated as a regular regression model input, was split into train and test sets using an 80/20 ratio. Random_state value was set to 42. The training dataset was split using 10-fold cross-validation with KFold (random_state set to 256). Our pipeline was also used on each split during CV. As a result, each fold contained predicted probabilities (predicted_proba) for each class in both binary and multiclass tasks, and the test set contained the corresponding averaged predict_proba value. These 72 features were then used as input to the regression model.

### 3.7. Leave-One-Gen-Out (LOGO) Experiment

The LOGO experiment employed the same feature selection methodology as previously described, excluding hyperparameter optimization for the final model. StandardScaler normalization was implemented to address feature scale heterogeneity, scaling both predictor variables and the efficacy target to zero mean and unit variance. Evaluation was focused on three representative target genes (SSB, SOD2, STAT1) using LGBM regression with RDKit-derived molecular descriptors, where categorical gene identifiers were numerically encoded via Label Encoder.

### 3.8. Visualization

All plots were constructed using Matplotlib 3.7.3, Seaborn 0.13.0 and Plotly 5.18.0 libraries on Python. The GC fraction was calculated using the corresponding function (gc_content()) in the BioPython library. The complementary lengths were calculated using the same library and the reverse_complement() function.

### 3.9. AI-Assisted Figure Generation

Figure 1 was generated using DALL-E 3 to create an initial schematic/illustration; the final figure was edited by the authors, who validated the scientific content for accuracy and consistency with the manuscript.

## 4. Conclusions

A highly efficient pipeline demonstrating SOTA performance in quantitative gene knockdown efficacy prediction across the full activity range, despite data imbalance, is presented for chemically modified siRNA. This pipeline integrates: (i) property matrices reflecting the chemical structure of modified nucleotides, (ii) LLM embeddings considering a long-range context of gene sequences, and (iii) a two-stage probability-enhanced model efficiently utilizing the results of classifiers’ training for correcting gene knockdown efficacy. For this purpose, several experiments were performed on the collected and curated dataset including (i) comprehensive data analysis revealing data completeness as well as the influence of chemical modifications on normalized gene knockdown efficacy, taking into account concentrations and incubation time, (ii) molecular descriptor screening with maximal performance in downstream tasks, (iii) LLM embeddings screening for optimal gene sequence representation, as well as (iv) ML model screening and probability-enhanced pipeline evaluation. We strongly believe these findings are not limited to this specific task and can serve as a unified pipeline for AI-assisted development of nucleic acid-based therapies.

## Figures and Tables

**Table 1:** Descriptors and ML models comparison in prediction tasks on normalized data.

Type	rdKit				PyBioMed				CDK			
Metrics	RMSE Train	RMSE Test	R^2^	CV R^2^	RMSE Train	RMSE Test	R^2^	CV R^2^	RMSE Train	RMSE Test	R^2^	CV R^2^
**LGBM**	10.56	15.57	0.711	0.681	10.61	15.37	0.717	0.682	10.07	15.44	0.716	0.676
**XGB**	8.18	15.77	0.704	0.652	8.38	16.32	0.683	0.650	8.04	16.17	0.688	0.649
**RF**	8.45	16.35	0.682	0.640	8.46	16.69	0.668	0.638	8.42	16.58	0.670	0.640

**Table 2:** Developed model comparison with existing solutions.

	Descriptors (Gene)	Descriptors (siRNA)	Model	RMSE	PCC (R)	R^2^
Current work	Mistral 7B embeddings	RDkit	Two-stage probability-enhanced LGBM-based approach	**12.27**	**0.91**	**0.84**
Liu et al., 2024 [38]	-	Property matrices	Cross-attention CNN	16.97	0.83	0.69
Dong et al., 2022 [39]	-	Property matrices (BCUT)	Partial least squares (PLS)	13.50	0.82	0.67
La Rosa et al., 2022 [40]	Graph nodes + k-mers		GNN (HinSAGE)	14.23	0.74	0.49
Dar et al., 2016 [41]	-	Mononucleotide composition	SVM	-	0.80	0.64
Murali et al., 2015 [42]	Energy scores		MLP	-	0.74	0.55

## Data Availability

Dataset preparation and code for models’ development available on github repository: https://github.com/GenerativeMolMachines/siRNA (accessed on 1 December 2025).

## Supplementary Materials

The following supporting information can be downloaded at: https://www.mdpi.com/article/10.3390/ijms262411791/s1.
