# Supplementary material for: Two-Stage Probability-Enhanced Regression on Property Matrices and LLM Embeddings Enables State-of-the-Art Prediction of Gene Knockdown by Modified siRNAs
Source: Int J Mol Sci. 2025 Dec 5;26(24):11791. doi: 10.3390/ijms262411791 (PMC12732354; doi:10.3390/ijms262411791)
Supplement: Supplementary file 1 [file ijms-26-11791-s001.zip › ijms-4008945-supplementary.pdf]

SUPPORTING INFORMATION

Two-Stage Probability-Enhanced Regression on Property Matrices and LLM Embeddings Enables State-of-the-Art Prediction of Gene Knockdown by Modified siRNAs

Ivan Golovkin [†], Denis Shatkovskii [†] and Nikita Serov *

Center for Artificial Intelligence in Chemistry, ITMO University, 191002 Saint-Petersburg, Russia;
golovkin2003@list.ru (I.G.); shatkovsky@scamt-itmo.ru (D.S.)
* Correspondence: serov@pish.itmo.ru
[†] These authors contributed equally to this work.

SUPPLEMENTARY TABLES

Table S1. Correlations of numerical variables with the target variable.

	**siRNA concentration**	**Duration after transfection**	**Efficacy, %**
**siRNA concentration**	1.0	0.029	-0.014
**Duration after transfection**	0.029	1.0	-0.109
**Efficacy, %**	-0.014	-0.109	1.0

Table S2. Correlations of categorical variables with the target variable.

	**Efficacy, %**
**Experiment of activity measurement**	0.062
**Cell/Organism type**	0.126
**Transfection method**	0.116

Table S3. Binary classification performance on the train and test set.

	Precision	Recall	F1-score	Accuracy	Matthews correlation coefficient (MCC)
**Train**	0.97	0.99	0.98	0.97	0.92
**Test**	0.92	0.93	0.92	0.90	0.77

Table S4. Multiclass classification performance on the test set. 1, 2, 3, and 4 stand for the ranges 0-23%, 27-47%, 52%-73%, and 77-100% knockdown efficacy, respectively.

	Class	Precision	Recall	F1-score
**Train**	1	**0.97**	0.95	0.96
	2	**0.95**	0.89	0.92
	3	0.95	0.92	0.94
	4	0.95	**0.99**	**0.97**
**Test**	1	0.75	0.73	0.74
	2	0.53	0.36	0.43
	3	0.62	0.65	0.63
	4	**0.81**	**0.89**	**0.85**

Table S5. Performance evolution of regressive ML models on unnormalized data during feature selection and hyperparameter optimization.

Stage	Train		Test	
	RMSE	R^2	RMSE	R^2
**All features**	11.29	0.85	17.11	0.65
**Top-100 features + Correlation threshold**	11.04	0.85	14.54	0.75
**Top-100 features + Correlation threshold + Optuna optimization**	**8.34**	**0.92**	**13.81**	**0.78**

Table S6. Performance comparison of LLM embeddings for binary classification of target genes.

	Precision	Recall	F1-score	Accuracy	Matthews correlation coefficient (MCC)
**Mistral**	**0.91**	0.94	0.92	0.90	0.77
**MistralDNA**	**0.91**	0.94	**0.93**	0.90	0.77
**HyenaDNA**	**0.91**	**0.95**	**0.93**	**0.91**	**0.79**

Table S7. Performance comparison of LLM embeddings for multiclass classification of target genes.

	Class	Precision	Recall	F1-score
**MistralDNA**	1	**<u>0.77</u>**	**<u>0.74</u>**	**<u>0.76</u>**
	2	0.51	0.39	0.44
	3	0.63	**<u>0.65</u>**	0.64
	4	**<u>0.82</u>**	0.88	**<u>0.85</u>**
**<u>Mistral</u>**	1	0.75	0.73	0.74
	2	0.53	**<u>0.36</u>**	**<u>0.43</u>**
	3	0.62	0.65	**<u>0.63</u>**
	4	0.81	**<u>0.89</u>**	**<u>0.85</u>**
**HyenaDNA**	1	0.71	0.73	0.72
	2	**<u>0.56</u>**	0.36	0.44
	3	**<u>0.71</u>**	0.60	0.65
	4	0.78	0.93	**<u>0.85</u>**

Table S8. Performance comparison of LLM embeddings for regression of target genes.

**Type**	**Mistral**			**MistralDNA**			**HyenaDNA**		
**Metrics**	**RMSE train**	**RMSE test**	**R^2 test**	**RMSE train**	**RMSE test**	**R^2 test**	**RMSE train**	**RMSE test**	**R^2 test**
**Value**	4.42	12.27	0.84	6.67	12.17	0.84	5.21	12.78	0.81

Table S9. Hyperparameters for LGBM models in different types of classification.

Parameter	Binary classification value	Multiclass classification value
boosting_type	dart	dart
num_leaves	127	143
learning_rate	0.309364356044555	0.38171180165144736
n_estimators	998	475
max_depth	-1	9
subsample	0.5559201794927165	0.6402853222406453
colsample_bytree	0.7469320659122414	0.7836079520669534
reg_alpha	0.8926731058854098	0.5171309709546598
reg_lambda	0.6832776414098194	0.6568004124243358

Table S10. Hyperparameters for LGBM regression models

Parameter	Regression value	Regression on the same split value as meta-learning approach
boosting_type	dart	dart
num_leaves	459	172
learning_rate	0.02	0.008
n_estimators	20000	3099
max_depth	6	8
subsample	0.4	0.7
colsample_bytree	0.3	0.4
reg_alpha	0.0024277041329914843	0.0010099559169945034
reg_lambda	9.98216025280689	0.29142712232160783
min_child_samples	15	20
min_data_per_groups	79	36
max_bin	97	148
min_child_weight	0.002477919474264366	0.07501787638307629
scale_pos_weight	8.842589967475636	6.026816484016717

Table S11. Leave-one-gene-out (LOGO) regression metrics for three example genes with various representations in the dataset.

Gene	R^2	RMSE	Samples count
SSB	0.60	14.64	120
SOD2	0.56	13.84	30
STAT1	0.28	29.97	48

Table S12. Designations of modifications and the corresponding SMILES representations of the modified nucleotide with adenine (A) modifications as an example.

№	Designation	**SMILES representation** (for modified A)
1.	2'-Methoxy-	Nc1ncnc2N(C3OC(COP(=O)(O)OP(=O)(O)OP(=O)(O)O)C(O)C(OC)3)cnc12
2.	Locked nucleic acid	Nc1ncnc2N(C3OC(C4)(COP(=O)(O)OP(=O)(O)OP(=O)(O)O)C(O)C(O4)3)cnc12
3.	2'-Deoxy-	Nc1ncnc2N(C3OC(COP(=O)(O)OP(=O)(O)OP(=O)(O)O)C(O)C3)cnc12
4.	2'-Fluoro-	Nc1ncnc2N(C3OC(COP(=O)(O)OP(=O)(O)OP(=O)(O)O)C(O)C(F)3)cnc12
5.	Unlocked nucleic acid	Nc1ncnc2N(C(CO)OC(COP(=O)(O)OP(=O)(O)OP(=O)(O)O)(CO))cnc12
6.	Inverted abasic	C3OC(CO)C(OP(=O)(O)OP(=O)(O)OP(=O)(O)O)C3(O)
7.	Hexitol nucleic acid	Nc1ncnc2N(C3COC(COP(=O)(O)OP(=O)(O)OP(=O)(O)O)C(O)C(O)3)cnc12
8.	4'-C-Hydroxymethyl Deoxyribonucleic acid	Nc1ncnc2N(C3OC(COP(=O)(O)OP(=O)(O)OP(=O)(O)O)(CO)C(O)C3)cnc12
9.	DeoxyThymidine	CC1=CN(C(=O)NC1=O)(C3OC(COP(=O)(O)OP(=O)(O)OP(=O)(O)O)C(O)C3)
10.	Altritol nucleic acid	Nc1ncnc2N(C3COC(CO)C(OP(=O)(O)OP(=O)(O)OP(=O)(O)O)C(O)3)cnc12
11.	2'-O-Aminoethyl-ribose	Nc1ncnc2N(C3OC(COP(=O)(O)OP(=O)(O)OP(=O)(O)O)C(O)C(OCCN)3)cnc12

12.	5’-Phosphate ribose	Nc1ncnc2N(C3OC(COP(=O)(O)OP(=O)(O)OP(=O)(O)O)C(O)C(O)3)cnc12
13.	Phosphorothioate	Nc1ncnc2N(C3OC(CO)C(O(P(=O)(S)O))C(O)3)cnc12
14.	2’-Hydroxy-	Nc1ncnc2N(C3OC(COP(=O)(O)OP(=O)(O)OP(=O)(O)O)C(O)C(O)3)cnc12
15.	4’-Thioribose	Nc1ncnc2N(C3SC(COP(=O)(O)OP(=O)(O)OP(=O)(O)O)C(O)C(O)3)cnc12
16.	2’-Aminoethoxymethyl-	Nc1ncnc2N(C3OC(COP(=O)(O)OP(=O)(O)OP(=O)(O)O)C(O)C(OCOCCN)3)cnc12
17.	2’-Aminopropoxymethyl-	Nc1ncnc2N(C3OC(COP(=O)(O)OP(=O)(O)OP(=O)(O)O)C(O)C(OCOCCCN)3)cnc12
18.	2’,4’-Carbocyclic-Ethylene-bridged nucleic acid-Locked nucleic acid	Nc1ncnc2N(C3OC(C4)(COP(=O)(O)OP(=O)(O)OP(=O)(O)O)C(O)C(C(O)C4)3)cnc12
19.	2’-Deoxy-2’-N,4-C-Ethylene-Locked nucleic acid	Nc1ncnc2N(C3OC(C4)(COP(=O)(O)OP(=O)(O)OP(=O)(O)O)C(O)C(N4)3)cnc12
20.	Triazole-linked nucleic acid	Nc1ncnc2N(CC(=O)N(CC)Cc1c[nH]nn1)cnc12
21.	Oxetane-Locked nucleic acid	Nc1ncnc2N(C34OC(COP(=O)(O)OP(=O)(O)OP(=O)(O)O)C(O)C(OC4)3)cnc12
22.	Serinol nucleic acid	Nc1ncnc2N(CC(=O)NC(CO)COP(=O)(O)OP(=O)(O)OP(=O)(O)O)cnc12
23.	5’-Cholesterol	CC(C)CCCC(C)C1CCC2C3CC=C4CC(O(C(=O)NCCOCCOCCOCCOCC(CO)OP(=O)(O)OP(=O)(O)OP(=O)(O)O))CCC4(C)C3CCC12C
24.	2’-O-methoxyethylribose	Nc1ncnc2N(C3OC(COP(=O)(O)OP(=O)(O)OP(=O)(O)O)C(O)C(OCCOC)3)cnc12
25.	2’-Guanidinoethyl-	Nc1ncnc2N(C3OC(COP(=O)(O)OP(=O)(O)OP(=O)(O)O)C(O)C(CC=NC(N)(N))3)cnc12
26.	2’-O-Benzyl-	Nc1ncnc2N(C3OC(COP(=O)(O)OP(=O)(O)OP(=O)(O)O)C(O)C(Oc4ccccc4)3)cnc12
27.	2’-Aminopropyl-	Nc1ncnc2N(C3OC(COP(=O)(O)OP(=O)(O)OP(=O)(O)O)C(O)C(OCCCN)3)cnc12
28.	2’-Cyanoethyl-	Nc1ncnc2N(C3OC(COP(=O)(O)OP(=O)(O)OP(=O)(O)O)C(O)C(OCC#N)3)cnc12
29.	Cyclohexenyl nucleic acid	Nc1ncnc2N(C1C=CC(CO)C(OP(=O)(O)OP(=O)(O)OP(=O)(O)O)C1)cnc12
30.	2,4-Difluorotoluene	Cc1ccc(F)cc1F

31.	3'-Amino-	Nc1ncnc2N(C3OC(COP(=O)(O)OP(=O)(O)OP(=O)(O)O)C(N)C(O)3)cnc12
32.	Deoxyadenine	Nc1ncnc2N(C3OC(COP(=O)(O)OP(=O)(O)OP(=O)(O)O)C(O)C3)cnc12
33.	2'-O-Guanidinopropyl-	Nc1ncnc2N(C3OC(COP(=O)(O)OP(=O)(O)OP(=O)(O)O)C(O)C(OCCC=NC(N)(N))3)cnc12
34.	5-Fluoro-2-Deoxyuridine	N1(C2OC(O)C(O)C2)C=C(F)C(=O)NC1=O
35.	5'-O-Methyl-	Nc1ncnc2N(C3OC(COC)C(OP(=O)(O)OP(=O)(O)OP(=O)(O)O)C(O)3)cnc12
36.	Inverted abasic, 2'-Methoxy-	C3OC(CO)C(OP(=O)(O)OP(=O)(O)OP(=O)(O)O)C3(OC)
37.	Inverted abasic, 2'-Deoxy-	C3OC(CO)C(OP(=O)(O)OP(=O)(O)OP(=O)(O)O)C3

SUPPLEMENTARY FIGURES

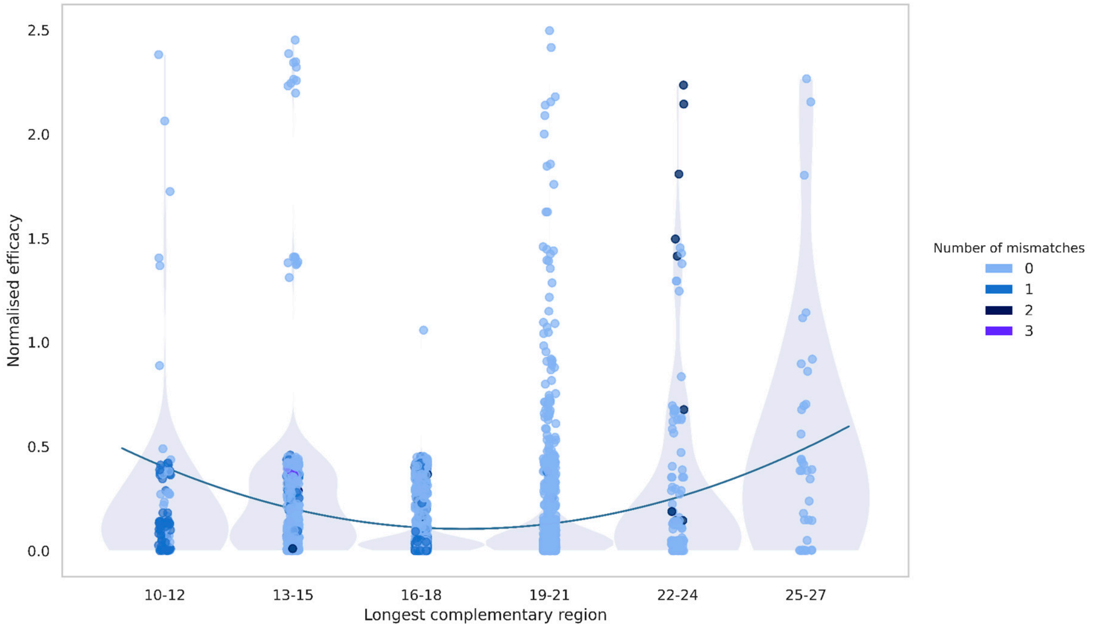

Figure S1. Distribution of normalized efficacy of sequences depending on the presence of mismatches with trendline inset (probability density plots shaded).

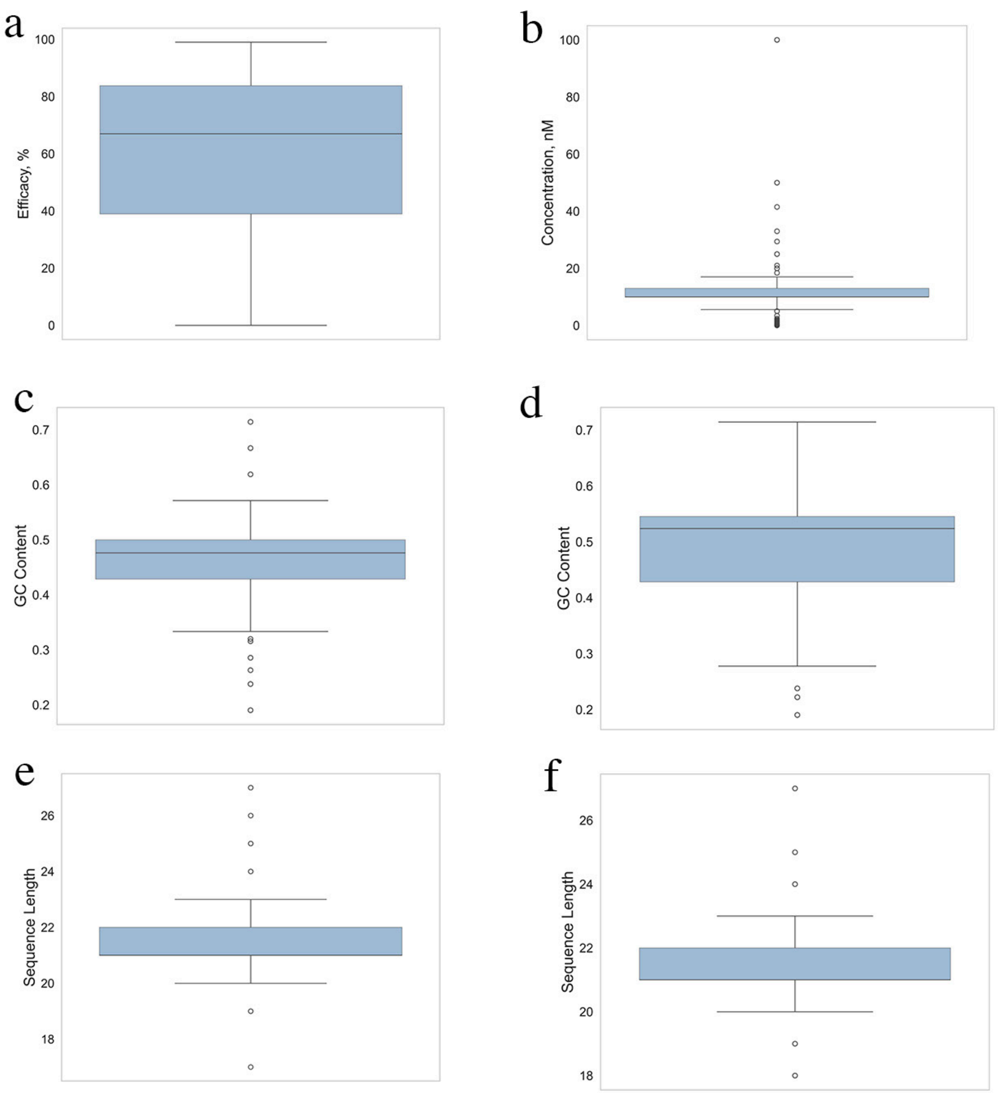

Figure S2. Boxplots for dataset variables: (**a**) efficacy, %, (**b**) siRNAs concentration, nM, (**c**) GC content of sense sequences, (**d**) GC content of antisense sequences, (**e**) sense sequences length, (**f**) antisense sequences length.
